# Supplementary material for: Ophthalmic Manifestations of the Monkeypox Virus: A Systematic Review and Meta-Analysis
Source: Pathogens. 2023 Mar 14;12(3):452. doi: 10.3390/pathogens12030452 (PMC10056031; doi:10.3390/pathogens12030452)
Supplement: Supplementary file 1 [file pathogens-12-00452-s001.zip › pathogens-2219361-supplementary figures S1-S4.pptx]

## Slide 1
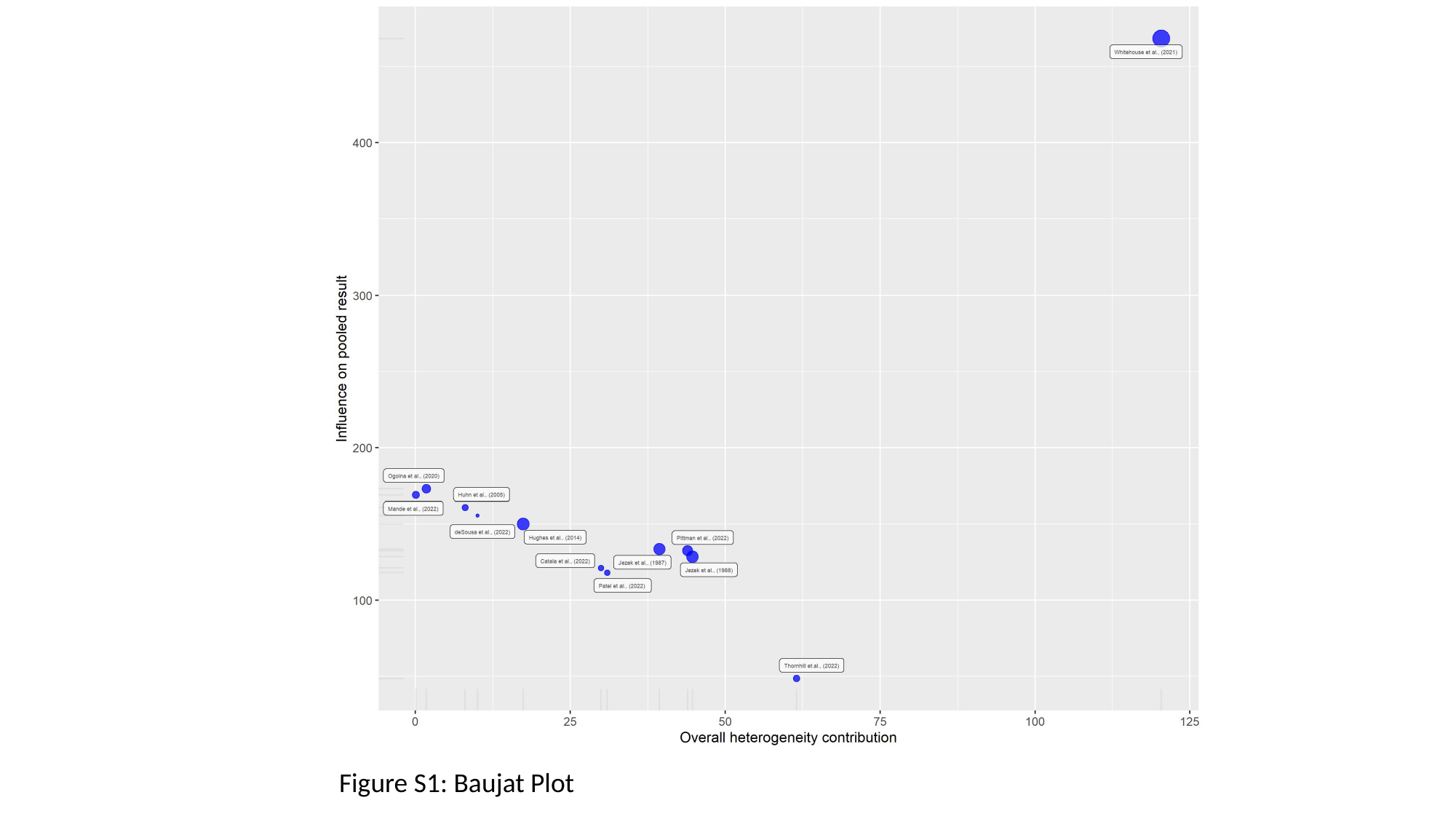

Figure S1: Baujat Plot

## Slide 2
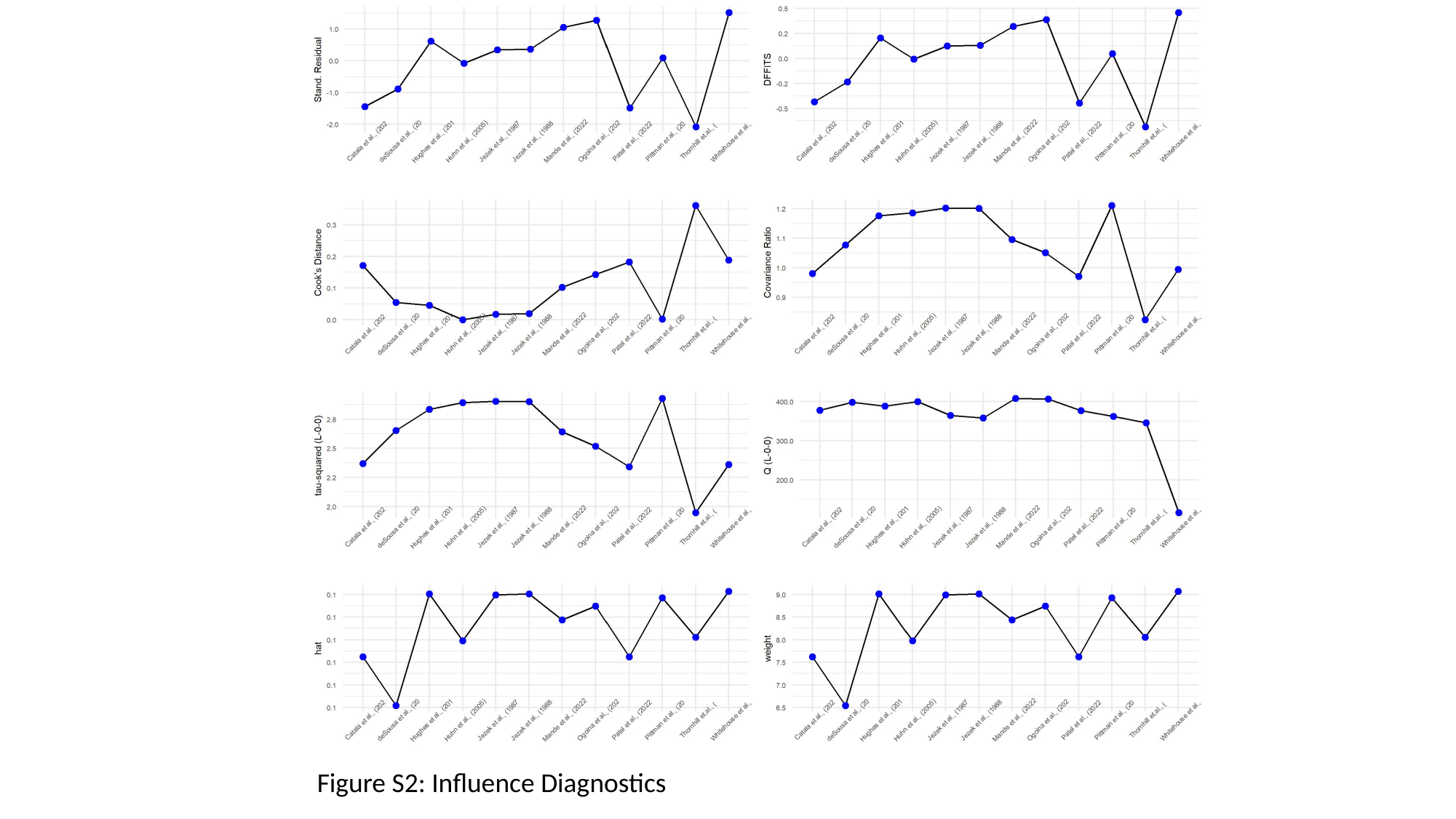

Figure S2: Influence Diagnostics

## Slide 3
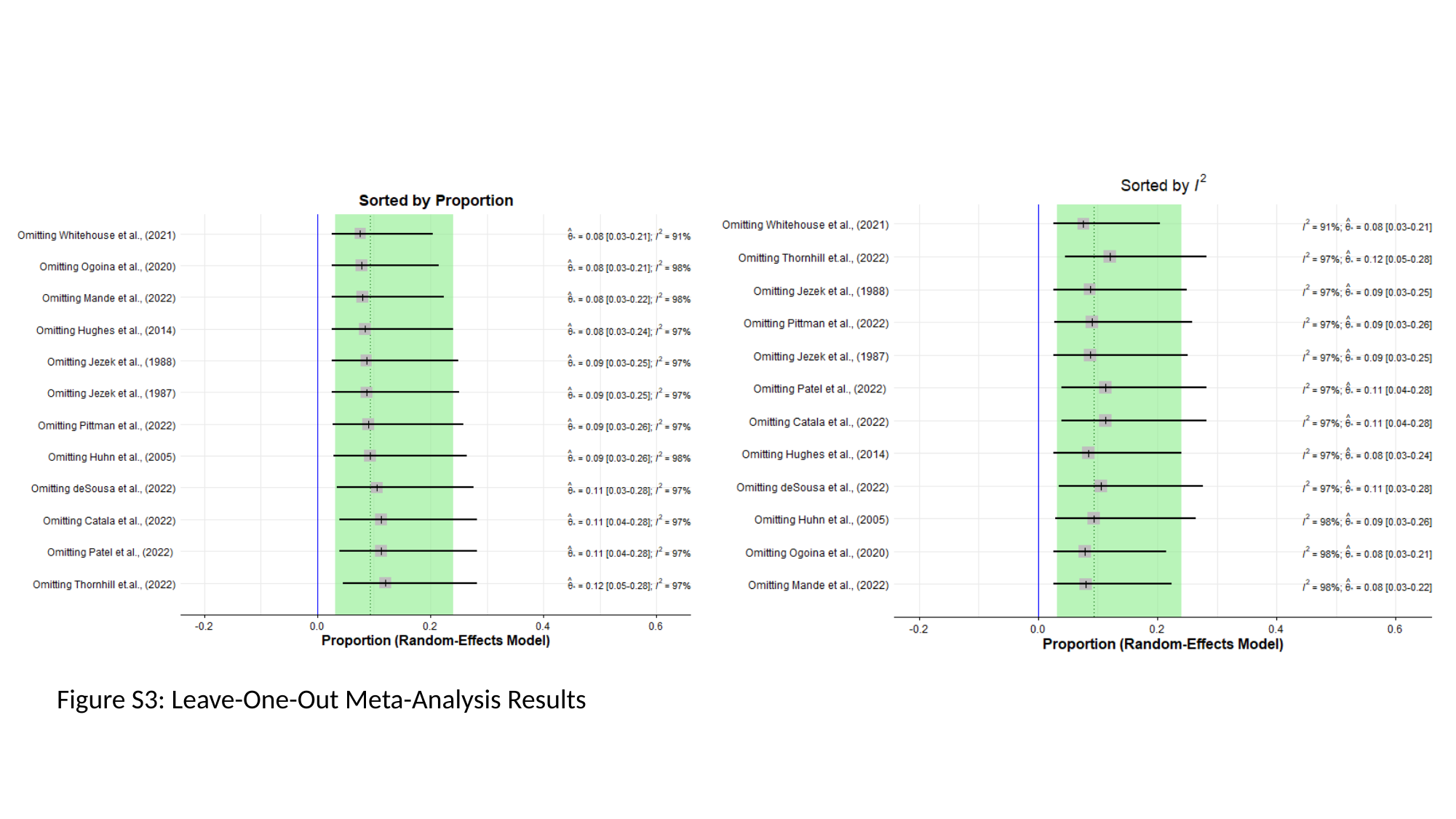

Figure S3: Leave-One-Out Meta-Analysis Results

## Slide 4
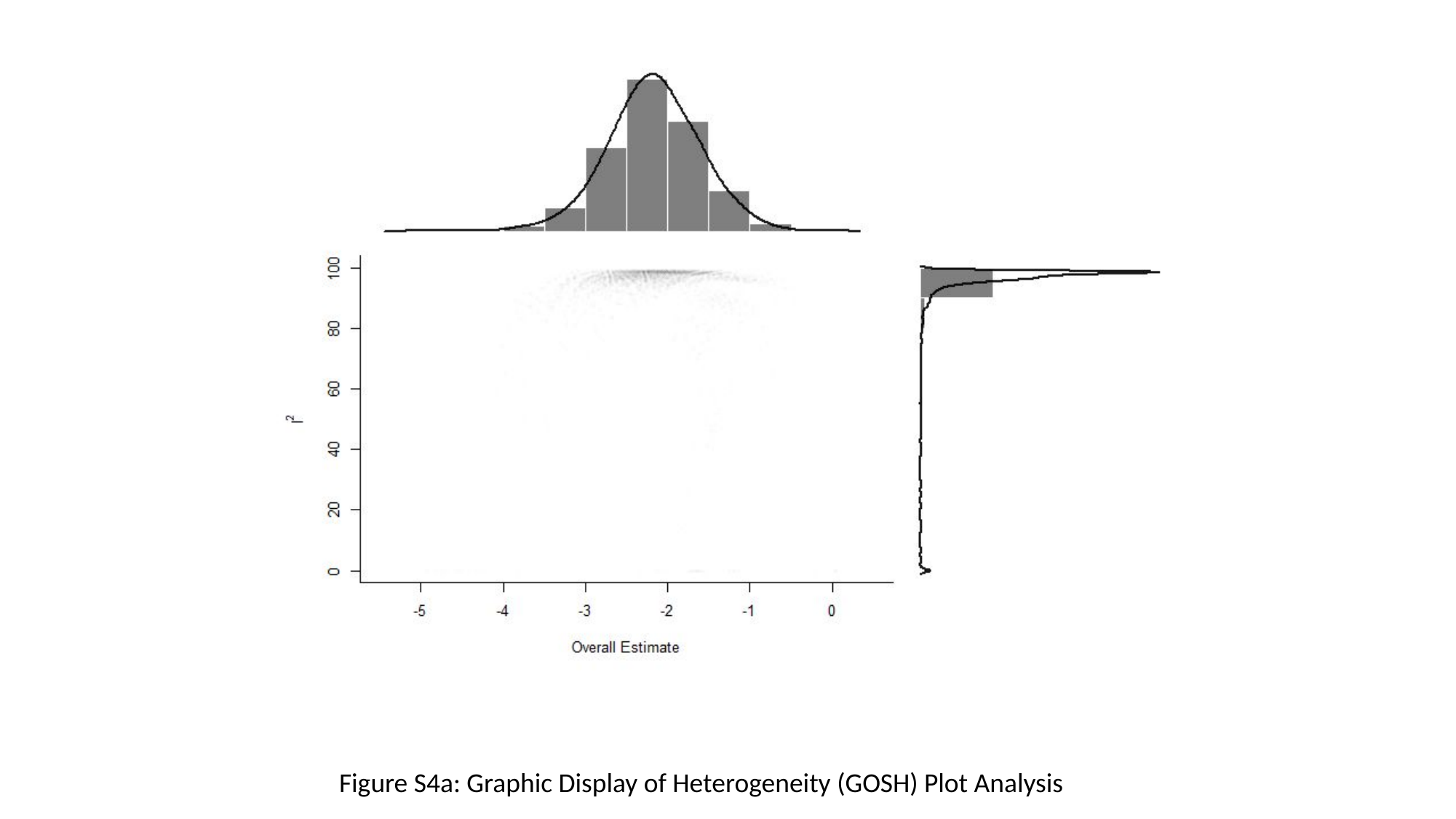

Figure S4a: Graphic Display of Heterogeneity (GOSH) Plot Analysis

## Slide 5
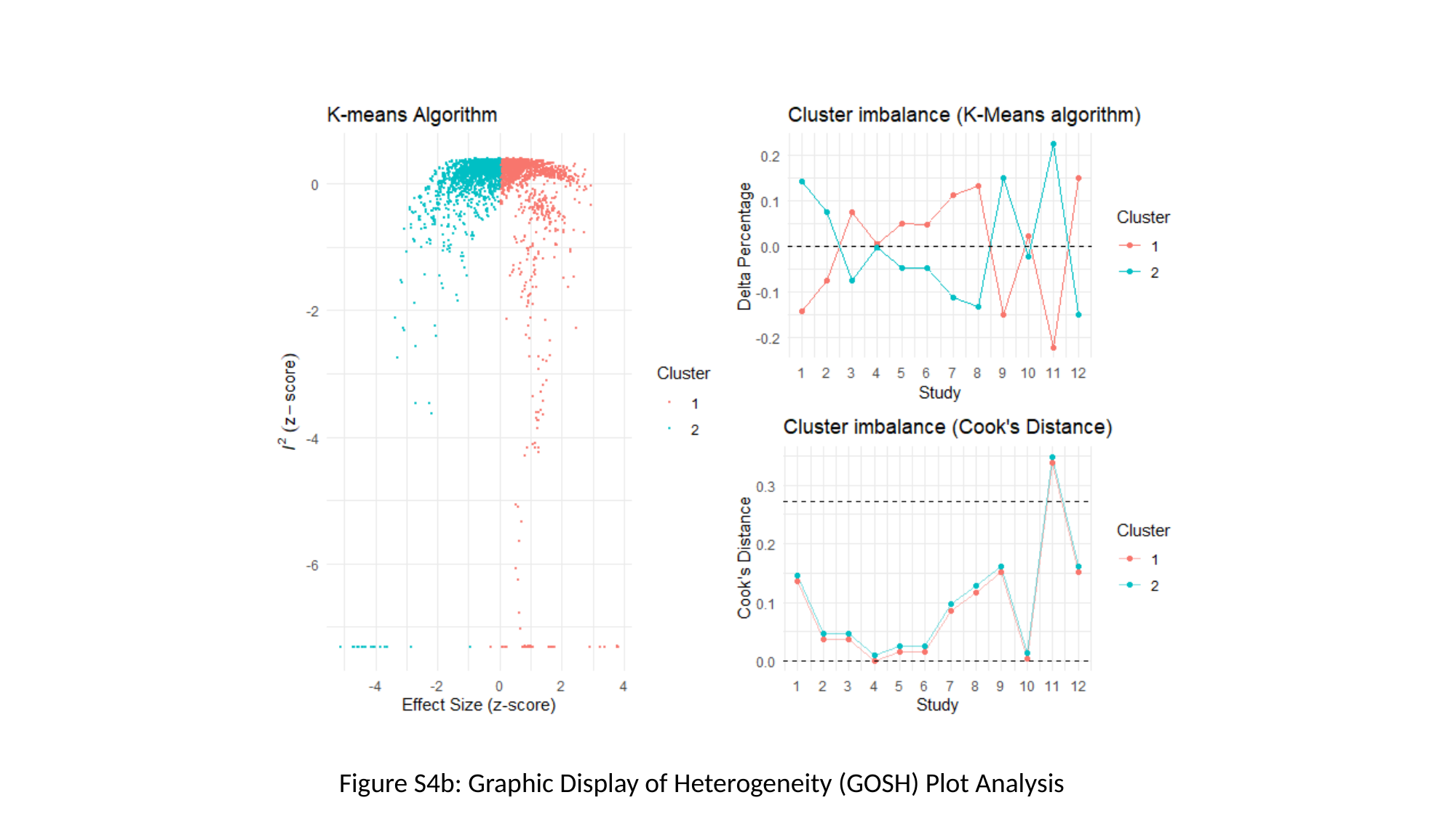

Figure S4b: Graphic Display of Heterogeneity (GOSH) Plot Analysis

## Slide 6
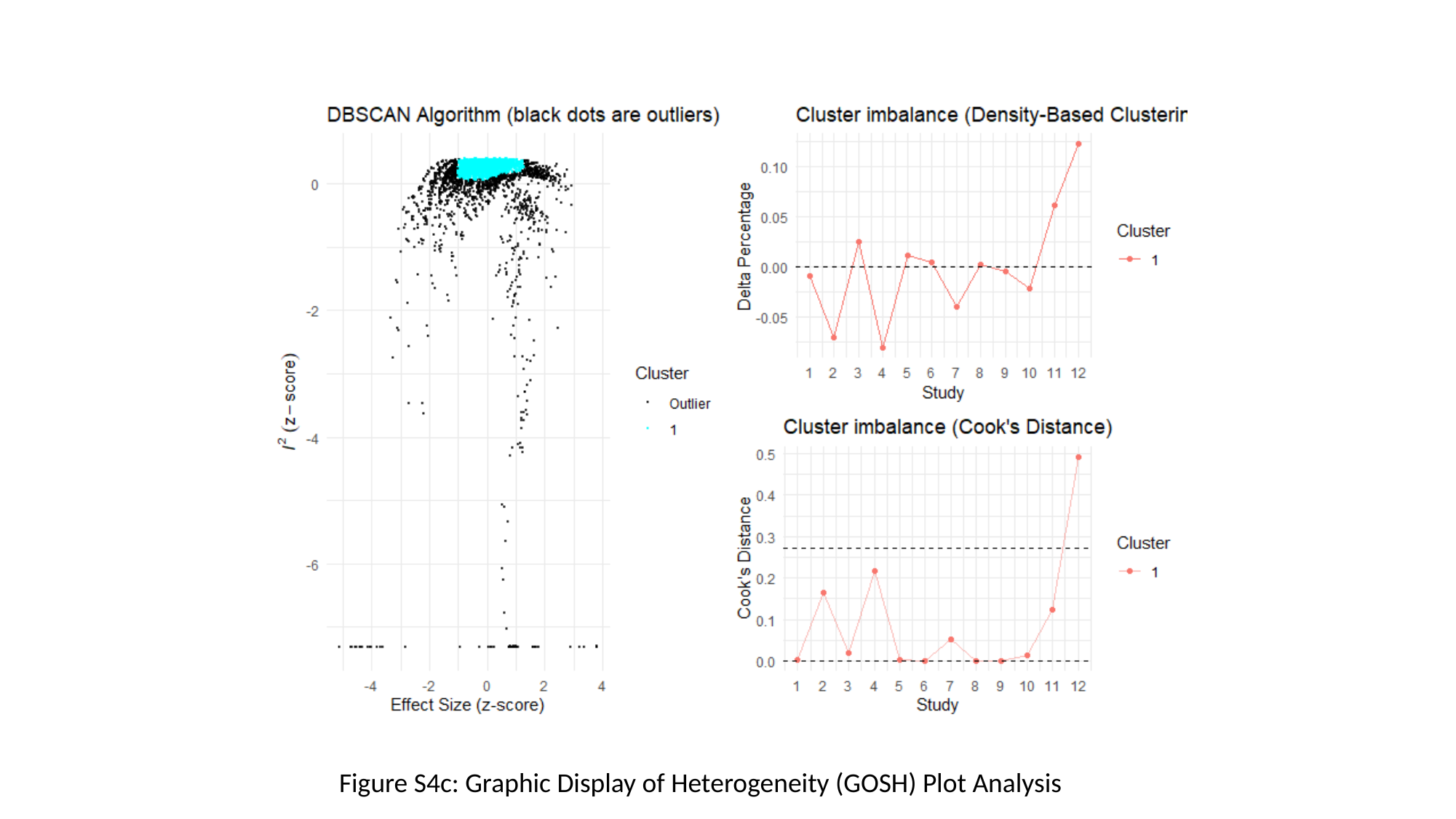

Figure S4c: Graphic Display of Heterogeneity (GOSH) Plot Analysis

## Slide 7
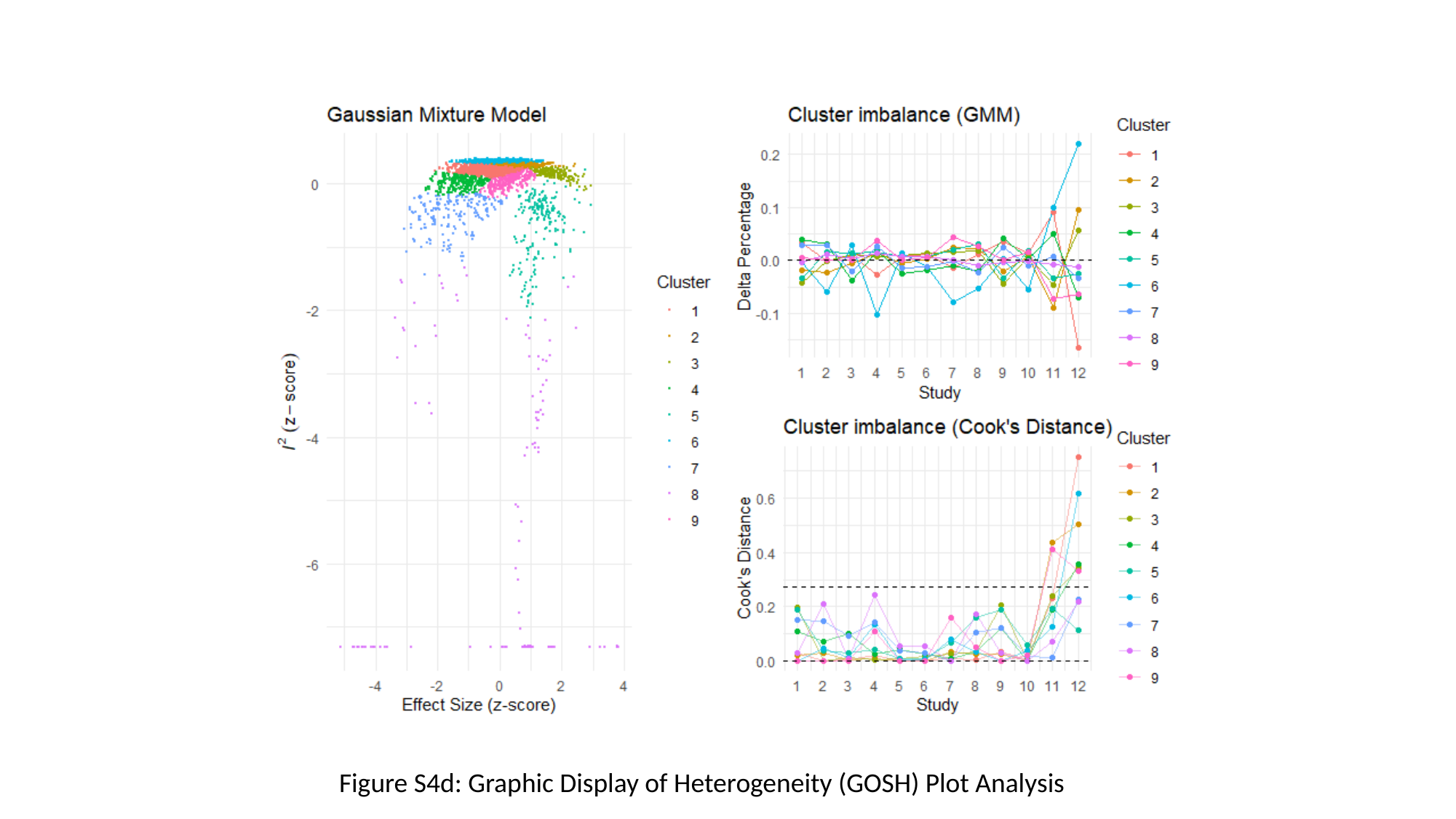

Figure S4d: Graphic Display of Heterogeneity (GOSH) Plot Analysis

## Slide 8
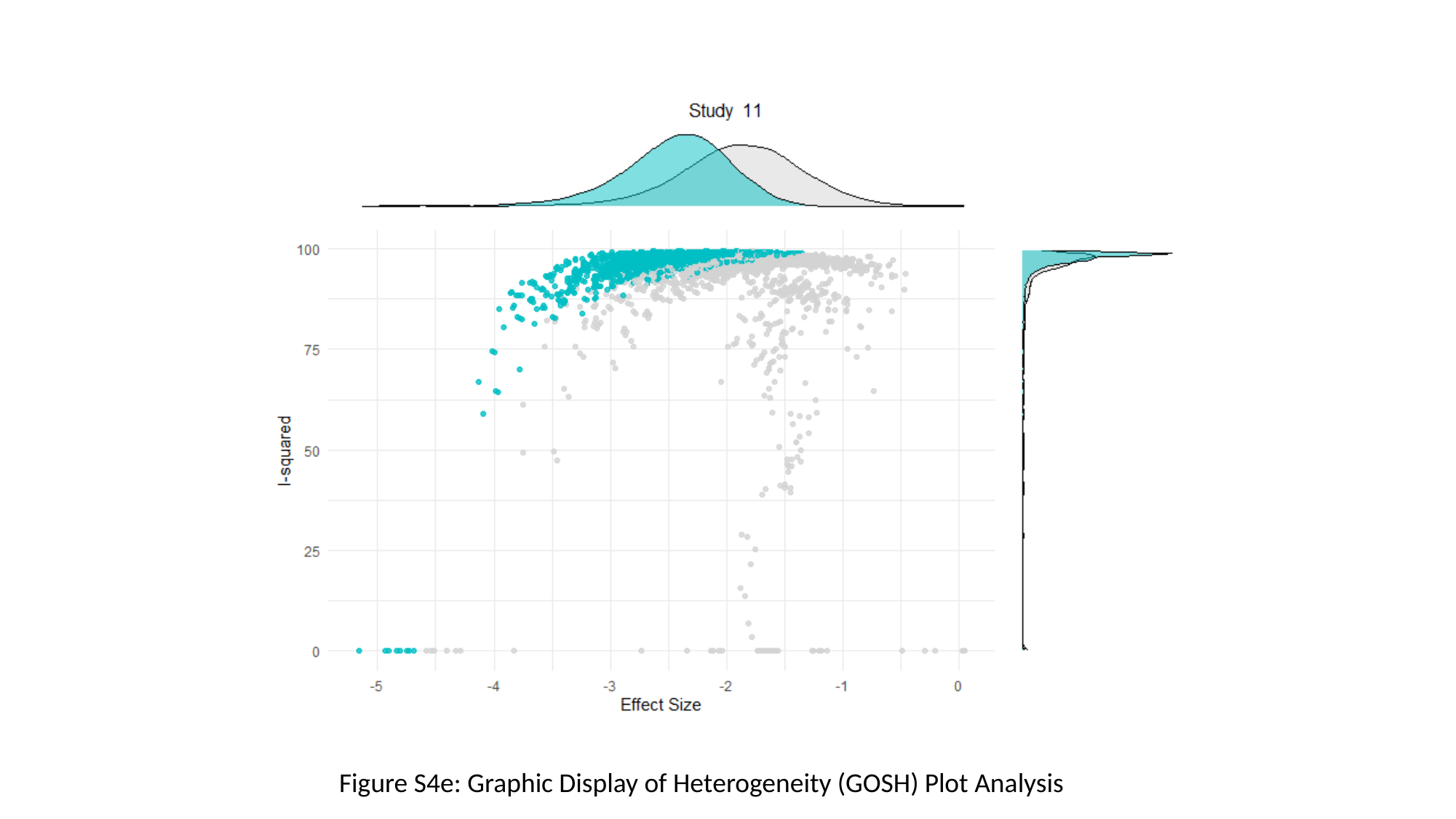

Figure S4e: Graphic Display of Heterogeneity (GOSH) Plot Analysis

## Slide 9
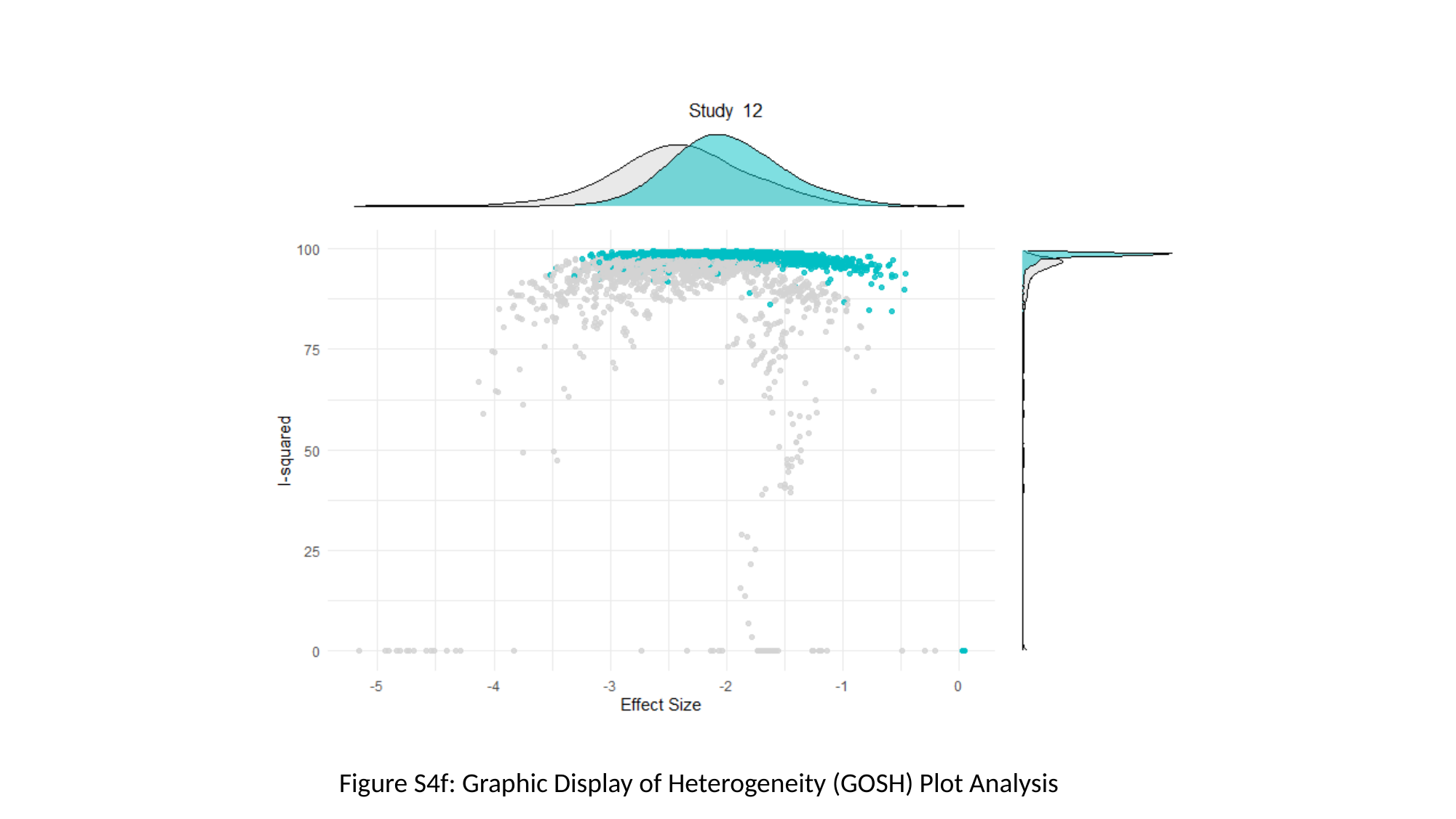

Figure S4f: Graphic Display of Heterogeneity (GOSH) Plot Analysis
